# High-speed high-resolution laser diode-based photoacoustic microscopy for in vivo microvasculature imaging

**DOI:** 10.1186/s42492-020-00067-5

**Published:** 2021-01-11

**Authors:** Xiufeng Li, Victor T C Tsang, Lei Kang, Yan Zhang, Terence T W Wong

**Affiliations:** Translational and Advanced Bioimaging Laboratory, Department of Chemical and Biological Engineering, Hong Kong University of Science and Technology, Hong Kong, China

**Keywords:** Photoacoustic microscopy, Laser diode, In vivo imaging, Microvasculature imaging

## Abstract

Laser diodes (LDs) have been considered as cost-effective and compact excitation sources to overcome the requirement of costly and bulky pulsed laser sources that are commonly used in photoacoustic microscopy (PAM). However, the spatial resolution and/or imaging speed of previously reported LD-based PAM systems have not been optimized simultaneously. In this paper, we developed a high-speed and high-resolution LD-based PAM system using a continuous wave LD, operating at a pulsed mode, with a repetition rate of 30 kHz, as an excitation source. A hybrid scanning mechanism that synchronizes a one-dimensional galvanometer mirror and a two-dimensional motorized stage is applied to achieve a fast imaging capability without signal averaging due to the high signal-to-noise ratio. By optimizing the optical system, a high lateral resolution of 4.8 μm has been achieved. In vivo microvasculature imaging of a mouse ear has been demonstrated to show the high performance of our LD-based PAM system.

## Introduction

Photoacoustic tomography is a hybrid imaging modality in which the detected ultrasonic signals are induced by the absorption of pulsed light [1]. Taking the advantage of intrinsic optical absorption contrast, photoacoustic microscopy (PAM) has been widely used to provide high-resolution and label-free images for functional, metabolic, and histological imaging [2–8]. However, the costly and bulky lasers, such as Q-switched diode-pumped solid-state laser, Ti:sapphire laser, or optical parametric oscillator laser, are usually required to generate high-energy light pulses with a short pulse width for PA signal generation, preventing the wide usage of PAM system for clinical applications. To address this issue, economical and compact laser diodes (LDs) have been investigated as alternative excitation sources in PAM [9–14]. However, most of them have limitations of low spatial resolution and/or low imaging speed. For example, a pulsed LD with a wavelength of 905 nm with a pulse energy of 3 μJ was used as the excitation source in PAM, achieving a lateral resolution of 7 μm [10]. Nevertheless, the pulse repetition rate is relatively low (1 kHz) and 128 times signal averaging is required to improve the signal-to-noise ratio (SNR), limiting the overall imaging speed. Recently, a laser scanning LD-based PAM system was developed without the need for signal averaging [12]. However, the image quality is not satisfactory because of the relatively low lateral resolution (21 μm).

Instead of using pulsed LDs, continuous-wave (CW) LDs, which can be overdriven by a pulse driver to generate high-energy light pulses, have also been proposed to be light sources in PAM. Compared to pulsed LDs, although the pulse energy of the pulse-overdriven CW LDs is relatively low (< 250 nJ), they are commercially available in a wide range of wavelengths, ranging from the visible to near-infrared wavelength regions, providing wavelengths that match the absorption peaks of various absorbers, thus, ensuring sufficient SNR in optical-resolution PAM. Among them, a 450 nm CW LD, which is pulse-driven by a custom-designed driver to generate light pulses with a pulse energy of ~ 200 nJ, pulsed width of ~ 10 ns, and repetition rate of 625 kHz, was applied to develop an LD-based PA mesoscope for vasculature imaging [13]. Although the repetition rate is high, the system requires 500 times signal averaging for mouse ear in vivo imaging. Another PA imaging system implementing with a compact fingertip 450 nm LD also suffers from low resolution and low imaging speed (256 times averaging) [14].

Therefore, there is still a challenge in implementing an LD-based PAM system that can generate high-resolution images with high imaging speed simultaneously. To this end, in this paper, we considered the size of the LD emitter while optimizing the optical system to focus the light onto samples, achieving a high lateral resolution of 4.8 μm. With a proper overdrive circuit implementation, high imaging speed is achieved with one-dimensional (1D) galvanometer mirror (GM) scanning that fully utilizes the high repetition rate (30 kHz) of the LD without the need for signal averaging.

## Method

**Fig. 1:**
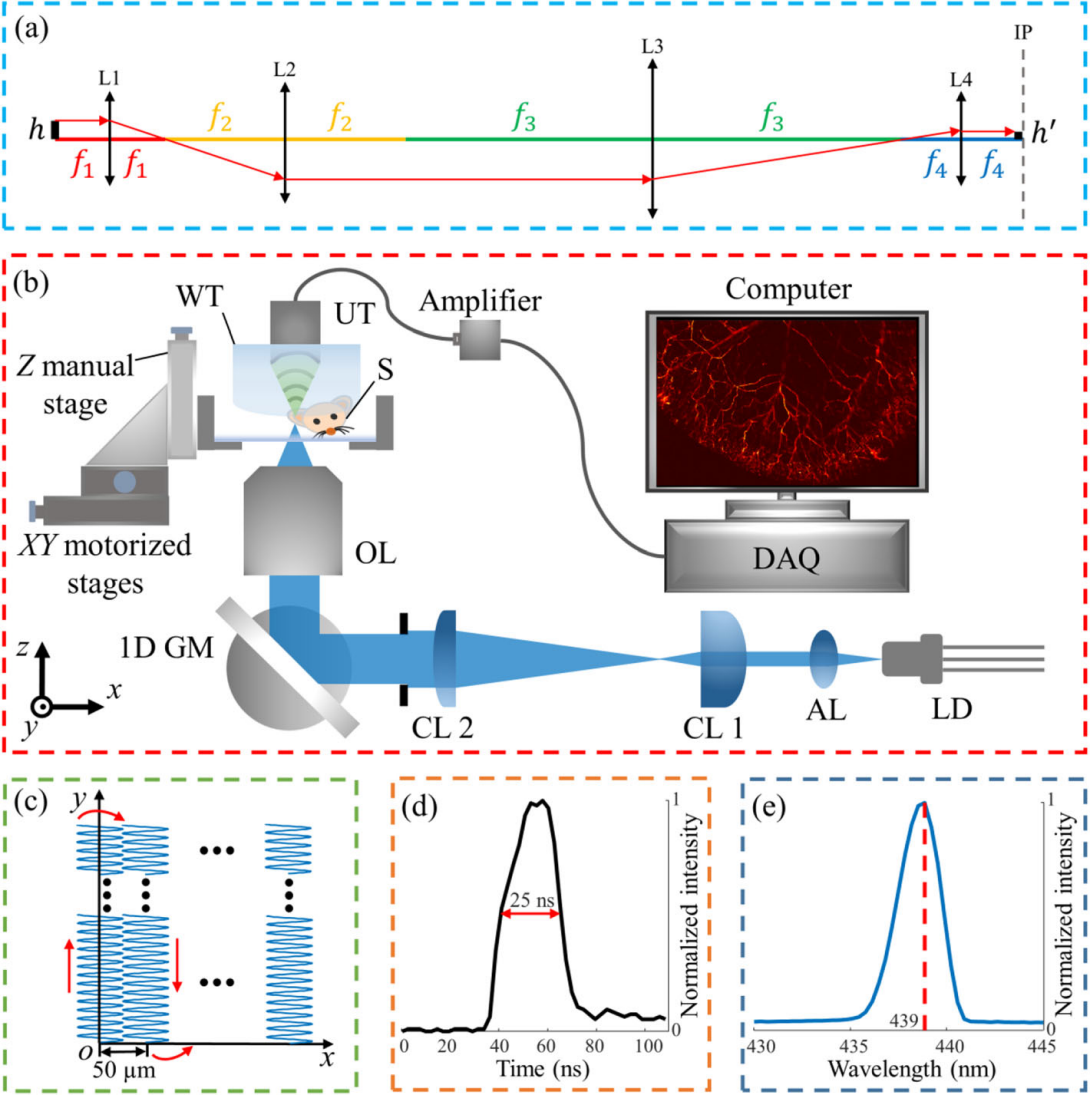
LD-based PAM system design. **a** The diagram of the optical system that demagnifies the emitter size of the LD on the image plane; **b** The schematic of LD-based PAM; **c** The scanning trajectory of the system; **d** The waveform of the LD light pulse, showing a pulse width of ~ 25 ns; **e** The spectrum of the LD light pulse, showing a center wavelength of ~ 439 nm. L: lens; f: focal length; IP: image plane; AL: aspheric lens; CL: cylindrical lens; OL: objective lens; S: sample; UT: ultrasonic transducer; WT: water tank; DAQ: data acquisition card

In this paper, a CW multimode blue LD (L450G1, Thorlabs, Inc.) is used as the excitation source. The light beam emitting from the LD is focused onto the sample by a set of lenses to excite PA signals. Different from solid-state lasers, which are usually considered as ideal light sources, the emitter size of LD has to be considered when designing a high-resolution LD-based PAM system. For the blue LD we employed, the emitter size has a dimension of 1 × 35 μm^2^ (vertical × horizontal). Therefore, to achieve high lateral resolution, the horizontal dimension is demagnified to prevent an elliptical focal spot. The optical system that we used to demagnify the size of the emitter on the image plane is shown in Fig. 1a. According to geometrical optics, the size of the image can be expressed as: *h*^′^ = (*f*_4_/*f*_3_) ∙ (*f*_2_/*f*_1_) ∙ *h*, where *f*_*i*_ is the focal length of the lens L_*i*_ (*i* = 1, 2, 3, 4), and *h* is the size of the object. Thus, by determining the ratio of (*f*_4_/*f*_3_) and (*f*_2_/*f*_1_), the size of the object on the image plane can be demagnified accordingly, leading to high-resolution imaging with the LD-based PAM system.

Based on the above analysis, we set up a high-speed and high-resolution LD-based PAM system as shown in Fig. 1b. The laser beam emitting from the LD is first collimated by an aspheric lens (A240TM-A, *f*_1_ = 8 mm, Thorlabs, Inc.). Since the LD has different beam divergence angle (30^o^ and 6^o^ in the vertical and horizontal directions, respectively), the laser beam is then expanded along the horizontal direction by a pair of cylindrical lenses (GCL-110114, *f*_2_ = 25 mm, Daheng Optics; LJ1267RM-A, *f*_3_ = 250 mm, Thorlabs, Inc.). An iris (~ 8 mm in diameter) is placed after the second cylindrical lens to control the light beam to a circular shape. The laser beam is then reflected by a 1D GM before it is focused by an objective lens (LMU-20X-UVB, *f*_4_ = 9.9 mm, Thorlabs, Inc.) onto the sample for PA signals excitation, achieving rapid scanning with a line-scanning interval of 50 μm on the sample. A hybrid scanning that synchronizes 1D optical and two-dimensional mechanical scanning is applied to achieve fast imaging for the whole sample [7, 15]. The laser-induced PA signals will be detected by a focused ultrasonic transducer (V324-SU, 25 MHz central frequency, Olympus NDT, Inc.), and amplified by two amplifiers (56 dB, two ZFL-500LN-BNC+, Minicircuit, Inc.). The signals are then filtered by a low-pass filter (BLP-70+, DC-60 MHz, Minicircuit, Inc.) before being collected by a data acquisition card (ATS9350, Alazar Technologies, Inc.). Finally, the signals are processed to reconstruct LD-based PAM images on a computer display.

The details about achieving high imaging speed using 1D GM can be found in our previous work [7]. In brief, as shown in the scanning trajectory (Fig. 1c), the 1D GM repeatedly reflects the incident laser pulses to scan samples along the *x*-axis with a line-scanning interval of ~ 50 μm, which is smaller than the acoustic focal spot of the ultrasonic transducer to maintain high detection sensitivity. The Y motorized stage [L-509.10SD00, PI (Physik Instrumente) Singapore LLP] is synchronized with the 1D GM and moves along the *y*-axis when the 1D GM finishes a line scan. After the Y motorized stage travels the preset distance, the X motorized stage [L-509.10SD00, PI (Physik Instrumente) Singapore LLP] moves along the *x*-axis with a step size of 50 μm. The Y motorized stage and the 1D GM system are then synchronized and scan again. The scanning process will stop when the entire sample is scanned completely.

Based on the above parameters of the optical lenses, the theoretical resolution can be estimated as *h*^′^ = (*f*_4_/*f*_3_) ∙ (*f*_2_/*f*_1_) ∙ *h* = (9.9/250) ∙ (25/8) ∙ 35 μm ≈ 4.3 μm. To generate light pulses, the blue CW LD is driven by a commercially-available pulse driver (PCO-7121, Directed Energy, Inc.) at a repetition rate of 30 kHz. The waveform of an emitted light pulse measured with a photodiode detector (PDA10A2, Thorlabs, Inc.) is shown in Fig. 1d, showing a pulse duration of ~ 25 ns. Figure 1e shows the spectrum of the emitted light measured with an optical spectrometer (USB 2000+, Ocean Optics, Inc.). The center wavelength of the laser is ~ 439 nm, which is shorter than that in the specification datasheet (center wavelength ~ 446 nm). The spectrum in the datasheet is measured in a CW operating condition with a power of ~ 3 W, while the power of the pulse-driven LD in this paper is < 5 mW. The observed spectral shift can be accounted for the differences in LD chip temperature as power differs between the CW operating mode and the pulse-driven mode.

## Results

### Resolution measurement

**Fig. 2:**
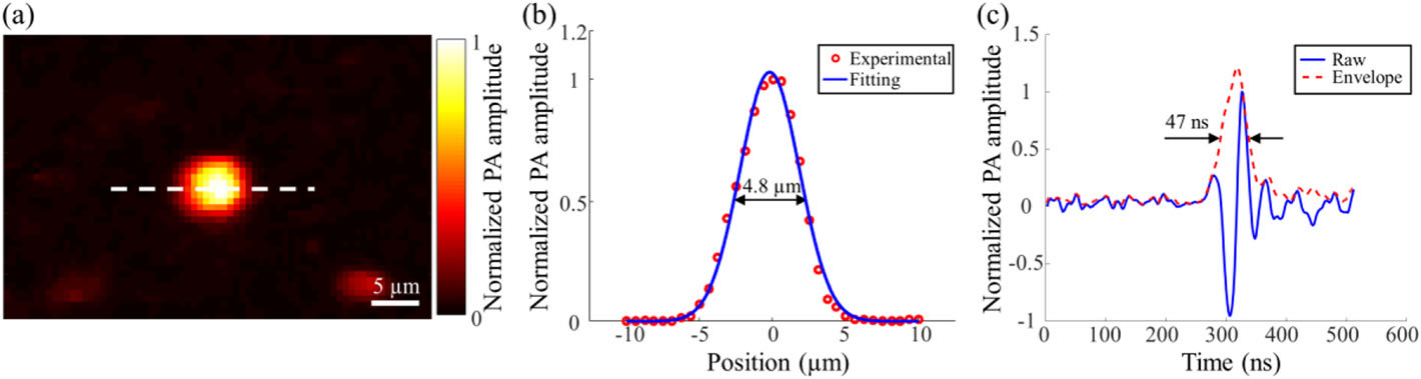
Spatial resolution measurement. **a** An LD-based PAM image of a carbon particle; **b** Lateral resolution measurement. The red circles are the experimental data along the white dotted line in Fig. **a**. The blue solid line is a Gaussian fitting function; **c** Axial resolution measurement. The red dotted line represents the envelope of the A-line signal of the central position of the carbon particle in Fig. **a**

To evaluate the spatial resolution of our LD-based PAM system, carbon particles with 1 μm in diameter were imaged. An LD-based PAM image of a particle is shown in Fig. 2a. To measure the lateral resolution, the experimental data along the white dotted line (Fig. 2a) was first plotted as the red circles in Fig. 2b and then fitted by a Gaussian function (blue solid line in Fig. 2b). The full width at half maximum (FWHM) of the fitted Gaussian function shows that the lateral resolution of our system is ~ 4.8 μm, which is close to the theoretical value (4.3 μm).

To measure the axial resolution, we applied the Hilbert transform to extract the envelope of the A-line PA signal of the carbon particle at the central position, shown in Fig. 2c. The FWHM of the envelope is ~ 47 ns, which represents ~ 70 μm in axial resolution by assuming the speed of sound to be ~ 1480 m/s.

### Leaf phantom imaging

**Fig. 3:**
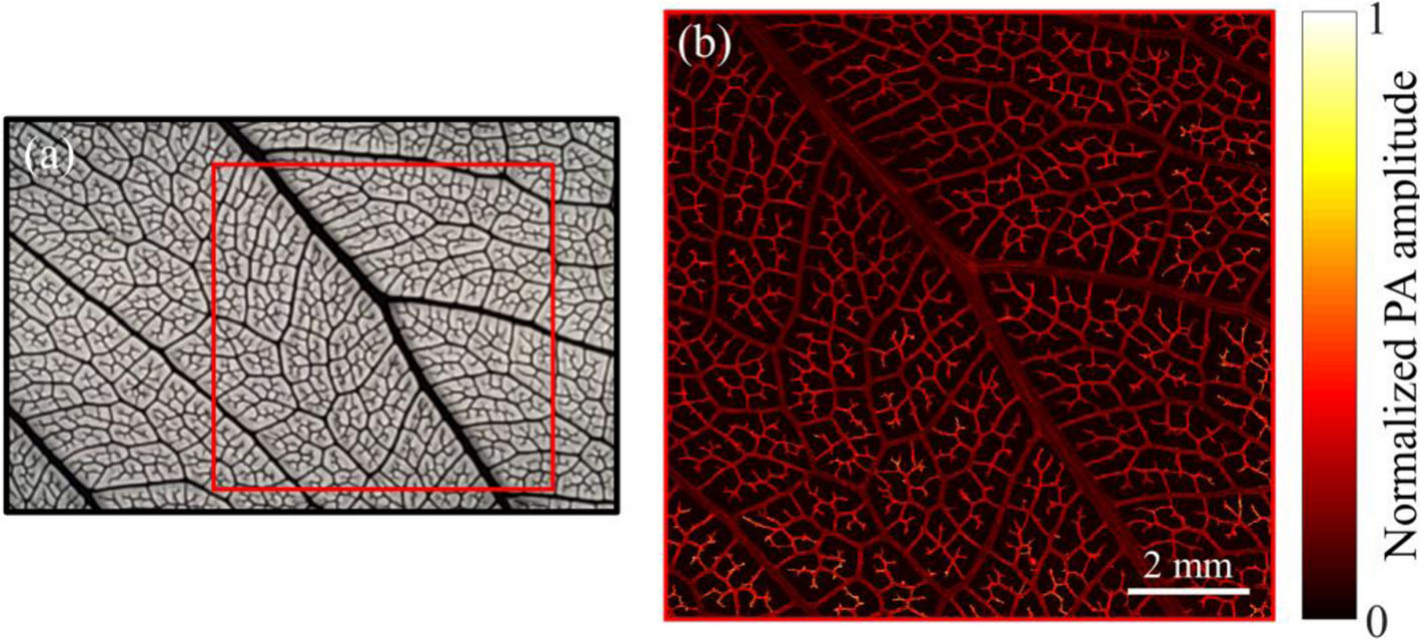
Leaf phantom imaging. **a** A photograph of a leaf phantom dyed with black ink; **b** An LD-based PAM image of the red marked region in Fig. **a**

To demonstrate the imaging capability of our system, a leaf phantom dyed with black ink was imaged. Figure 3a shows a photograph of the leaf phantom, and Fig. 3b shows the LD-based PAM image of the red marked region in Fig. 3a. We can see from Fig. 3 that the structure of the leaf in the LD-based PAM (Fig. 3b) image shows high similarity with that in the photograph (Fig. 3a). The maximum SNR can reach up to ~ 42 dB with a pulse energy of ~ 30 nJ. The field-of-view (FOV) of the LD-based PAM image (Fig. 3b) is 10 × 10 mm^2^. With this superior SNR, no signal averaging is required. With the high repetition rate (30 kHz) of the LD and fast scanning mechanism, the total imaging time for this large FOV is ~ 28.5 minutes with a scanning step size of 1.3 μm in the *x*-axis, and 1.6 μm in the *y*-axis (corresponding to 32 points on each line-scanning interval using GM scanning). According to our current lateral resolution (~ 4.8 μm), a larger scanning step can be set to further improve the imaging speed without sacrificing the imaging resolution.

### In vivo microvasculature imaging of a mouse ear

**Fig. 4:**
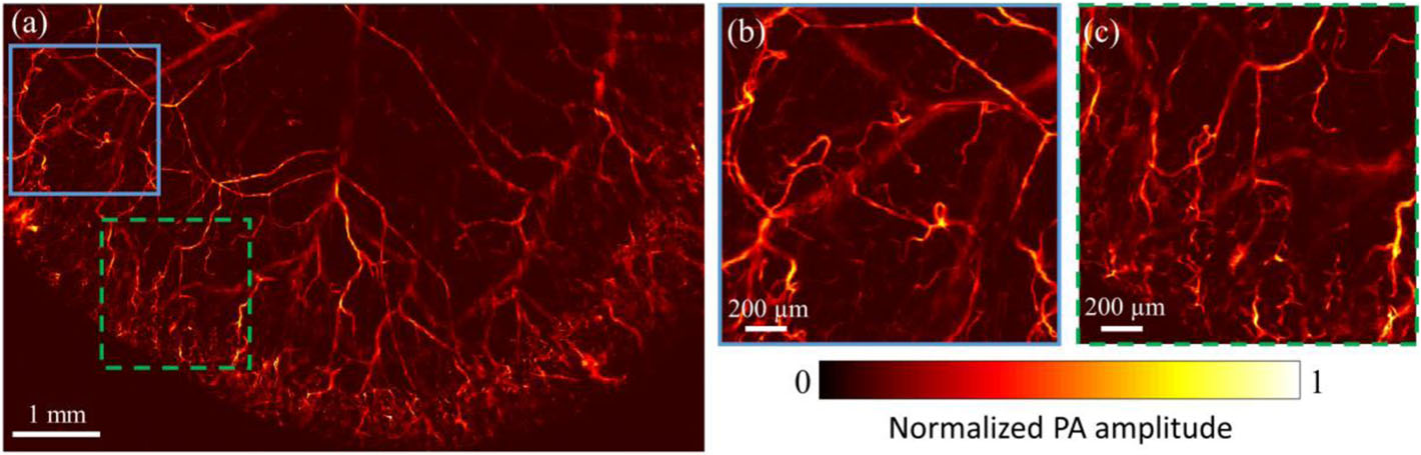
**a** In vivo LD-based PAM imaging of a mouse ear. **b** and **c** Close-up images of the blue and green marked regions in Fig. **a**

To verify the potential of our LD-based PAM system for in vivo microvasculature imaging, a mouse ear was imaged. The protocol of animal experiments was approved by the Animal and Plant Care Facility at The Hong Kong University of Science and Technology. The mouse was maintained anesthetized with 2% isoflurane mixed with oxygen at a flow rate of 0.8 L/min during the experiment. An LD-based PAM image of the mouse ear acquired by our system is shown in Fig. 4a, where the distribution of microvasculature networks can be clearly observed. The SNR can reach up to 31 dB with a pulse energy of ~ 60 nJ. The optical fluence on the tissue surface is ~ 0.8 mJ/cm^2^, which is lower than the American National Standards Institute safety limit of 20 mJ/cm^2^ [16]. The FOV is 8 × 5 mm^2^. The image acquisition time is ~ 11.5 minutes. Figures 4b and c show the close-up images of the blue and green marked regions in Fig. 4a, respectively. The microvasculature can be clearly resolved. These promising results evaluate that our LD-based PAM system can provide high-quality images for in vivo microvasculature imaging at high speed.

## Discussions and conclusions

In this paper, we developed a high-resolution and efficient LD-based PAM system by optimizing the parameters of the optical system design according to the emitter size of the LD. Moreover, the high SNR allows no signal averaging, and thus a fast optical scanning mechanism can be used to improve the overall imaging speed. To the best of our knowledge, it is the first demonstration of an LD-based PAM system that can achieve high resolution (< 5 μm) and high imaging speed (30 kHz A-line rate) simultaneously. Nevertheless, the performance of our system can be further improved. First, the focal lengths of the two-cylindrical lenses are relatively large, which is less suitable for a more compact system. A pair of cylindrical lenses with shorter focal lengths, while maintaining the equivalent focal length ratio can be used. Second, the current system is in transmission mode, which limits its applications to only imaging thin tissue samples, e.g., mouse ear in vivo (Fig. 4). To promote the LD-based PAM system with more applications, a ring-shaped ultrasonic transducer [17] or other optically transparent ultrasonic transducers [18] can be used to develop a reflection-mode LD-based PAM system. Third, the size of the pulse driver can be further minimized to make the system more compact. A compact, low-cost, and high-performance LD-based PAM system will be beneficial for various biomedical applications, and is expected to be more accessible for clinical imaging.

The stability of the pulse energy of pulse-driven CW LDs has been investigated in ref. [13]. We also tested our LD-based PAM system for more than 24 hours under a repetition rate of 30 kHz with a pulse energy of 100 nJ (on the sample plane). No damage or degradation of optical energy has been observed, proving the high stability of the pulse-driven CW LD.

In conclusion, we developed a high-speed and high-resolution LD-based PAM system for microvasculature imaging. The light beam is tightly focused on the sample by an optimized demagnifying optical system, achieving a high lateral resolution of 4.8 μm. Our system does not require any signal averaging, thus, enabling high imaging speed with the integration of a 1D GM. The in vivo mouse ear imaging demonstrated that our system could provide high-quality images for microvasculature at high imaging speed.

## Data Availability

The datasets used and analyzed during the current study are available from the corresponding author on reasonable request.

## Abbreviations

PAM: Photoacoustic microscopy
LD: Laser diode
SNR: Signal-to-noise ratio
CW: Continuous wave
1D: One-dimensional
GM: Galvanometer mirror
FWHM: Full width at half maximum
FOV: Field-of-view
